# PIK3CA-Mutant Non-Small Cell Lung Cancer Refractory to Two Lines of Chemoimmunotherapy: A Case Report

**DOI:** 10.7759/cureus.100648

**Published:** 2026-01-02

**Authors:** Takayuki Higashi, Tomoyuki Araya, Toshiyuki Kita, Ryo Hara, Hazuki Takato

**Affiliations:** 1 Respiratory Medicine, National Hospital Organization (NHO) Kanazawa Medical Center, Kanazawa, JPN

**Keywords:** adenosquamous carcinoma, immune checkpoint inhibitor, non-small cell lung cancer (nsclc), pik3ca mutation, poor prognosis

## Abstract

*PIK3CA* mutations play a critical role in tumorigenesis by driving constitutive activation of the phosphoinositide 3-kinase (PI3K) signaling pathway. These mutations have been identified in a small subset of non-small cell lung cancer (NSCLC), but their clinical significance and response to chemoimmunotherapy remain unclear. A 44-year-old man with a 24-pack-year smoking history presented with hoarseness and exertional dyspnea. Imaging demonstrated an irregular left upper-lobe pulmonary nodule consistent with the primary lesion, along with metastatic involvement of the left frontal lobe, cervical-mediastinal lymph nodes, right kidney, and lumbar vertebrae. Endobronchial ultrasound-guided transbronchial needle aspiration of the station 4R lymph node confirmed adenosquamous carcinoma. Molecular analysis revealed a *PIK3CA* E545K mutation without coexisting oncogenic driver alterations. The programmed cell death ligand 1 tumor proportion score indicated low expression (1-9%). Despite first-line carboplatin, nab-paclitaxel, and pembrolizumab followed by second-line carboplatin, pemetrexed, nivolumab, and ipilimumab, the disease progressed rapidly, and the patient died four months after diagnosis. This case illustrates that isolated *PIK3CA*-mutant NSCLC can be highly refractory to conventional chemoimmunotherapy, including regimens incorporating a cytotoxic T-lymphocyte-associated antigen 4 inhibitor. Further clinical investigation of PI3K-targeted therapies is warranted to establish effective treatment strategies for *PIK3CA*-mutant NSCLC.

## Introduction

Lung cancer remains one of the most frequently diagnosed malignancies worldwide [1]. Although targeted therapies and immunotherapy have dramatically improved survival, certain subsets of patients still experience poor outcomes due to a lack of established standard treatments [2].

Phosphoinositide 3-kinases (PI3Ks) are a family of lipid kinases that phosphorylate intracellular phosphoinositides, thereby regulating key cellular processes such as proliferation, survival, and metabolism [3,4]. PI3Ks are classified into three major classes, such as class I, class II, and class III, based on their structural and functional characteristics [3,4]. Class I PI3Ks are obligate heterodimers and are subdivided into two subclasses, class IA and class IB [3,4]. Class IA PI3Ks consist of one of the catalytic subunits, p110α, p110β, or p110δ, in complex with a p85-type regulatory subunit and are activated primarily by receptor tyrosine kinases [3,4]. By contrast, class IB PI3Ks consist of the p110γ catalytic subunit in complex with either the p101 or p84/p87 regulatory subunit and are activated by G-protein-coupled receptors [3,4].

Among the catalytic isoforms, p110α, encoded by *PIK3CA*, is the most frequently mutated and plays a critical role in tumorigenesis [4]. *PIK3CA *mutations occur predominantly at E542K and E545K in exon 9 and at H1047R in exon 20, resulting in constitutive activation of the PI3K signaling pathway [4,5]. These mutations are detected in approximately 2-5% of non-small cell lung cancers (NSCLCs) and have been associated with poor prognosis [6-9].

PI3K-targeted therapies, such as alpelisib, have demonstrated clinical benefit in *PIK3CA*-mutant breast cancer and have been approved by the US Food and Drug Administration [10]. However, their efficacy in NSCLC has not been established. Furthermore, the therapeutic outcomes of conventional chemotherapy and chemoimmunotherapy in *PIK3CA*-mutant NSCLC remain poorly understood. We report a case of *PIK3CA*-mutant NSCLC that was refractory to two different regimens of chemoimmunotherapy, ultimately resulting in rapid disease progression and death within four months of diagnosis.

This article was previously presented as a meeting abstract at the 2025 Annual Meeting of the Japan Lung Cancer Society on November 6, 2025.

## Case presentation

A 44-year-old man presented with a one-month history of hoarseness and exertional dyspnea. He had no significant past medical history. His family history was notable for breast cancer in his sister and malignant lymphoma in his grandmother. He had a 24-pack-year smoking history and worked as a plasterer. Physical examination revealed multiple firm, immobile lymph nodes in the left cervical region, and his vital signs were stable.

Laboratory findings showed a white blood cell count of 12,000/μL, a C-reactive protein level of 11.81 mg/dL, and elevated serum tumor markers, including carcinoembryonic antigen at 54.2 ng/mL and cytokeratin 19 fragment at 7.0 ng/mL (Table 1).

**Table 1:** Baseline laboratory findings Laboratory evaluation revealed elevated WBC, CRP, and D-dimer levels, as well as increased serum tumor markers, including CEA and CYFRA. No laboratory evidence of organ dysfunction, including hepatic or renal impairment, was identified, and electrolyte levels were within normal limits. ALB: albumin; ALP: alkaline phosphatase; ALT: alanine aminotransferase; AST: aspartate aminotransferase; BAS: basophil; BUN: blood urea nitrogen; CEA: carcinoembryonic antigen; CL: chloride; CRP: C-reactive protein; CR: creatinine; CYFRA: cytokeratin 19 fragment; EOS: eosinophil; EGF: estimated glomerular filtration rate; HB: hemoglobin; HT: hematocrit; K: potassium; LYM: lymphocyte; LDH: lactate dehydrogenase; NA: sodium; NEU: neutrophil; PLT: platelet; PROGRP: pro-gastrin-releasing peptide; RBC: red blood cell; T-BIL: total bilirubin; TP: total protein; UA: uric acid; WBC: white blood cell

Parameter (unit)	Value	Reference range
WBC (/μL)	12,000	4,500-9,000
Neu (%)	76.4	38-74
Lym (%)	13.1	16.5-49.5
Mon (%)	9.6	5-10
Eos (%)	0.7	0-10
Bas (%)	0.2	0-2
RBC (×10⁴/μL)	476	382-500
Hb (g/dL)	14.7	11.7-14.6
Ht (%)	43.9	34.3-44.2
Plt (×10⁴/μL)	12.1	15.8-34.8
CRP (mg/dL)	11.81	0-0.4
T-Bil (mg/dL)	0.8	0.3-1.2
TP (g/dL)	7.3	6.7-8.3
ALP (U/L)	332	38-113
AST (U/L)	15	13-33
ALT (U/L)	12	6-27
LDH (U/L)	238	119-229
Alb (g/dL)	3.4	4.0-5.0
Na (mEq/L)	137	135-149
K (mEq/L)	4.3	3.5-4.9
Cl (mEq/L)	98	96-108
BUN (mg/dL)	7.6	8-22
Cr (mg/dL)	0.61	0.5-0.8
eGFR (mL/min/L)	112.5	60-100
UA (mg/dL)	4.3	2.3-7.0
D-dimer (μg/mL)	1.9	0-1
CEA (ng/mL)	54.2	0-5
CYFRA (ng/mL)	7	0-3.5
ProGRP (pg/mL)	36.2	0-81

**Figure 1:**
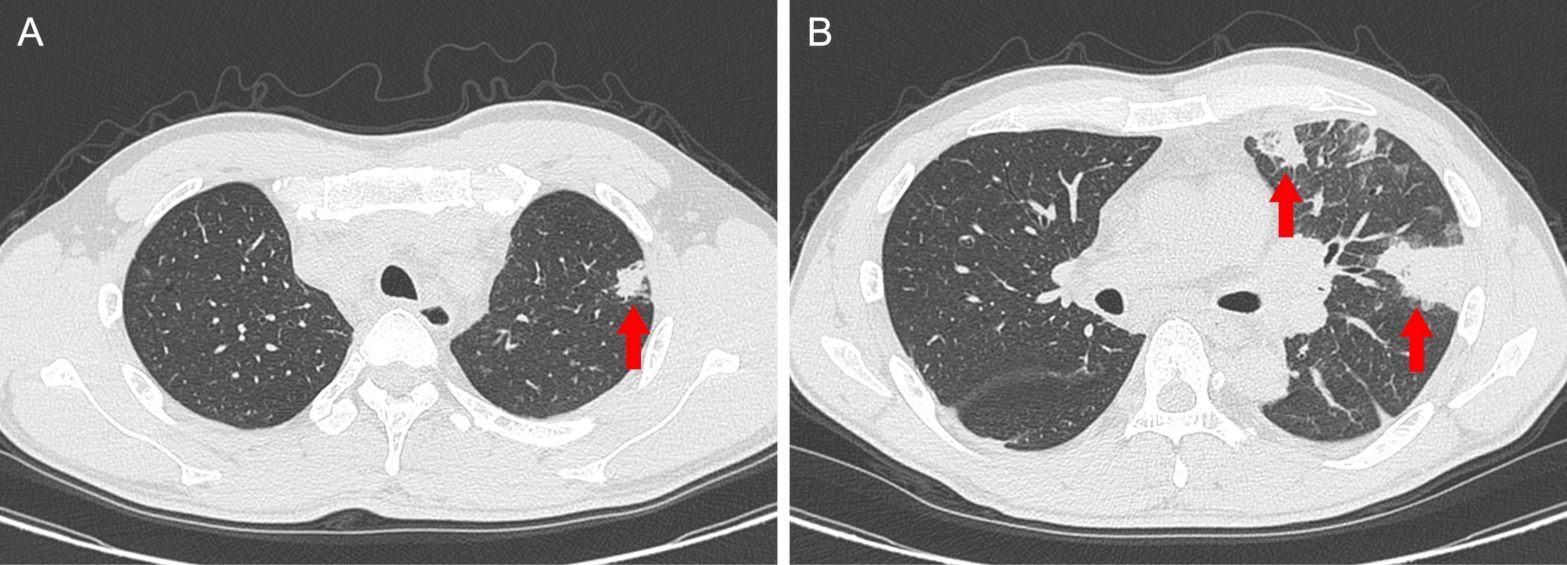
CT findings Chest CT shows an irregularly shaped nodule in the left upper lobe (A, arrow), accompanied by multiple wedge-shaped opacities in the left lung parenchyma (B, arrows).

**Figure 2:**
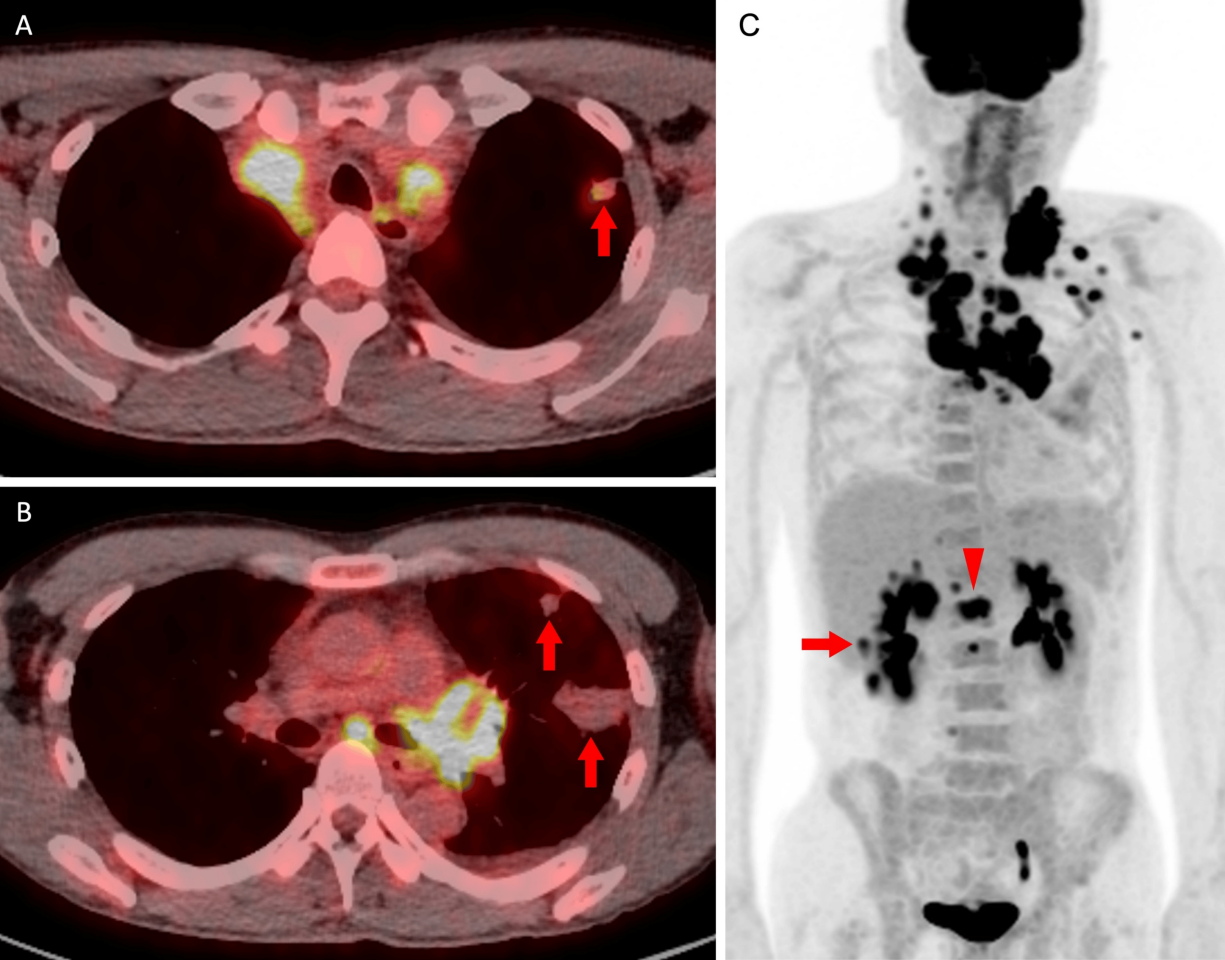
18F-FDG PET/CT findings 18F-FDG PET/CT shows intense FDG uptake in the enlarged lymph nodes (A-C) and moderate uptake in the pulmonary nodule (A, arrow), right kidney (C, arrow), and lumbar vertebrae (C, arrowhead), while no abnormal FDG accumulation is observed in the wedge-shaped opacities in the left lung (B, arrows). FDG: fluorodeoxyglucose

**Figure 3:**
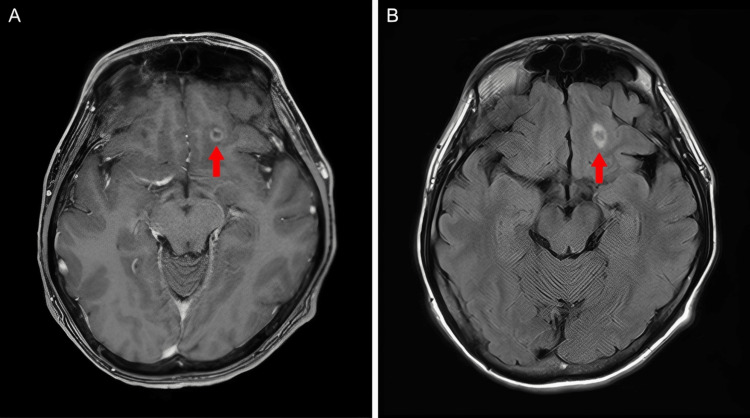
MRI findings Contrast-enhanced T1-weighted imaging demonstrated a ring-enhancing lesion in the left frontal lobe (A, arrow). FLAIR imaging showed perilesional edema associated with the lesion (B, arrow). FLAIR: fluid-attenuated inversion recovery

Cervical and chest CT demonstrated an irregular nodule in the left upper lobe and multiple enlarged lymph nodes extending from the cervical to the mediastinal regions. Additionally, multiple wedge-shaped opacities were observed in the left lung parenchyma (Figure 1). 18F-fluorodeoxyglucose (FDG) PET/CT revealed intense FDG uptake in the enlarged lymph nodes and moderate uptake in the pulmonary nodule, right kidney, and lumbar vertebrae. In contrast, the wedge-shaped opacities in the left lung showed no abnormal FDG accumulation (Figure 2). Contrast-enhanced brain magnetic resonance imaging demonstrated a ring-enhancing lesion in the left frontal lobe (Figure 3).

**Figure 4:**
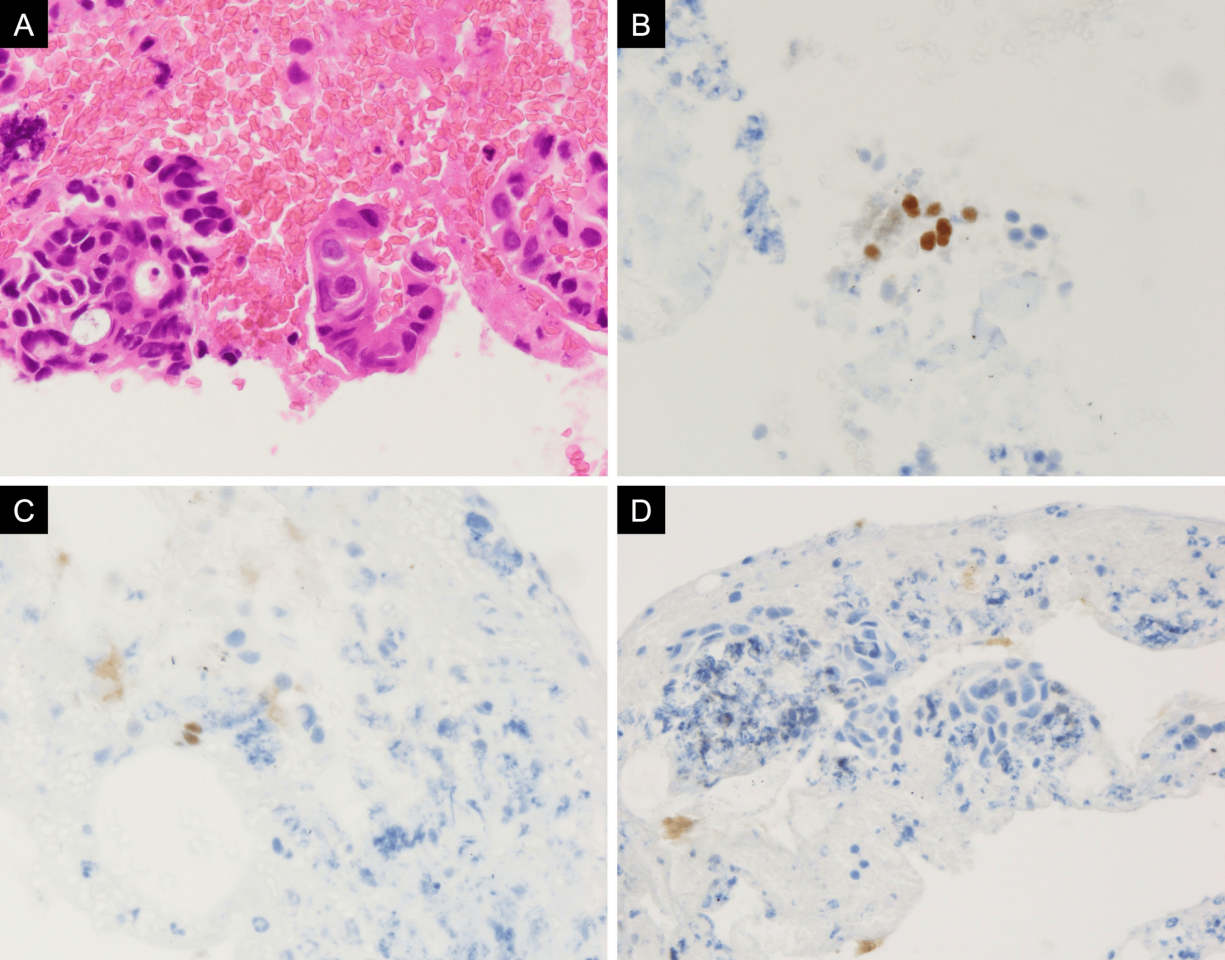
Pathological findings of the station 4R lymph node Hematoxylin and eosin staining revealed both glandular formation and keratinization (A, ×600). Immunohistochemistry showed focal positivity for p40 (B, ×600) and thyroid transcription factor-1 (C, ×600) and negativity for Napsin A (D, ×400).

Endobronchial ultrasound-guided transbronchial needle aspiration of the station 4R lymph node confirmed adenosquamous carcinoma. Immunohistochemical staining showed focal positivity for thyroid transcription factor-1, p40, and cytokeratin 5/6, and negativity for Napsin A, consistent with adenosquamous differentiation (Figure 4). The Oncomine Dx Target Test identified a *PIK3CA *E545K mutation, whereas no alterations were detected in *EGFR*, *BRAF*, *HER2 (ERBB2)*, *ALK*, *ROS1*, *MET*, or *RET*. The cobas^®^ EGFR Mutation Test v2 was also negative. The programmed cell death ligand 1 tumor proportion score indicated low expression (1-9%). Tumor mutational burden and microsatellite instability were not assessed.

The left lung nodule was considered the primary lesion, whereas the lesions in the brain, lymph nodes, bones, and kidney were consistent with metastatic disease. The clinical stage was cT2N3M1c, corresponding to stage IVB. Tracheal stenosis and bilateral recurrent laryngeal nerve palsy, attributed to tumor invasion and compression, were noted. Additionally, the wedge-shaped opacities in the left lung were interpreted as pulmonary infarction. The patient’s Eastern Cooperative Oncology Group performance status (ECOG PS) was 1.

Because no approved PI3K-targeted therapy for NSCLC was available and no applicable clinical trial could be identified, PI3K inhibitors were not administered. First-line treatment with carboplatin, nab-paclitaxel, and pembrolizumab was initiated. However, disease progression occurred after two cycles, with the emergence of a new right adrenal metastasis and enlargement of preexisting lymph node lesions. Given the patient’s young age, stable ECOG PS of 1, and limited exposure to first-line therapy, a multidisciplinary team conference recommended an immune checkpoint inhibitor (ICI)-containing second-line regimen. Accordingly, treatment with carboplatin, pemetrexed, nivolumab, and ipilimumab, a cytotoxic T-lymphocyte-associated antigen 4 (CTLA-4) inhibitor, was initiated. However, airway obstruction continued to worsen, and the patient’s ECOG PS declined to 3 after the first cycle. Therefore, the best supportive care was selected, and palliative radiotherapy (30 Gy in 10 fractions) to the neck and mediastinum was administered, leading to transient symptomatic improvement. Nevertheless, the patient died two months later, approximately four months after the initial diagnosis.

## Discussion

This case highlights that *PIK3CA*-mutant NSCLC can be refractory to both ICIs and cytotoxic chemotherapy. Notably, our patient was a young male smoker with adenosquamous carcinoma who exhibited rapid progression of lymph node disease.

The disease progressed rapidly despite two platinum-based chemoimmunotherapy regimens, including one incorporating a CTLA-4 inhibitor. Previous studies have suggested that *PIK3CA *mutations promote immune evasion by remodeling the tumor microenvironment into an immunosuppressive state through activation of the PI3K signaling pathway, which recruits myeloid-derived suppressor cells and suppresses cytotoxic CD8+ T-cell activity. This mechanism attenuates antitumor immunity and renders tumors refractory to ICIs [11]. In addition, *PIK3CA*-mutant cancers, such as breast and colorectal cancers, have been shown to be refractory to cytotoxic chemotherapy [12,13]. A similar tendency may be observed in *PIK3CA*-mutant NSCLC, although clinical evidence remains limited.

Previous studies have indicated that *PIK3CA*-mutant NSCLC is more likely to present with lymph node involvement, suggesting that activation of the PI3K signaling pathway may promote lymphatic invasion and metastatic potential of tumor cells [9,14].

*PIK3CA* mutations frequently coexist with other canonical oncogenic driver alterations, such as *EGFR* or *KRAS *[8,15]. In contrast, our patient harbored an isolated activating *PIK3CA *E545K mutation without concurrent oncogenic driver alterations. In a retrospective cohort of *PIK3CA*-mutant advanced NSCLC, isolated *PIK3CA *mutations were reported to occur more frequently in older patients with squamous histology, and the median overall survival was 12 months [16]. By comparison, our patient was relatively young, had adenosquamous carcinoma, and died approximately four months after diagnosis. This atypical case expands the clinical spectrum of isolated *PIK3CA*-mutant NSCLC. Several studies have reported that isolated *PIK3CA*-mutant NSCLC exhibits a poorer prognosis than cases with co-occurring oncogenic driver alterations [14,15]. This unfavorable outcome may be explained by the lack of effective standard treatment options for NSCLC harboring isolated *PIK3CA* mutations, whereas NSCLC with other oncogenic driver alterations may benefit from targeted therapy [15].

Although no standard targeted therapy has been established for isolated *PIK3CA*-mutant NSCLC, emerging evidence suggests that PI3K-targeted therapies may have activity in a specific molecular subset [16]. In other *PIK3CA*-mutant malignancies, PI3K inhibitors have demonstrated clinical benefit [10], and preclinical studies provide further rationale for a range of PI3K-targeted approaches. For example, bladder cancer models have shown that combined PI3K inhibition and immune checkpoint blockade creates an immunostimulatory tumor microenvironment and produces strong synergistic antitumor effects [17]. Additionally, endometrial and breast cancer models have shown that multi-node inhibition targeting PI3K and downstream signaling components achieves more complete pathway suppression than single-node inhibition [18]. Collectively, these findings suggest that PI3K-targeted therapies warrant further clinical evaluation as potential treatment options for *PIK3CA*-mutant NSCLC.

## Conclusions

This case highlights that isolated *PIK3CA*-mutant NSCLC may be refractory to conventional chemoimmunotherapy, including regimens incorporating a CTLA-4 inhibitor. Further clinical investigation of PI3K-targeted therapies, ideally in prospective basket or umbrella trials and in larger real-world cohorts, is warranted to establish effective treatment strategies for *PIK3CA*-mutant NSCLC.
